# Atypical presentation of H1N1-induced thrombotic microangiopathy with CD46 gene mutation 

**DOI:** 10.5414/CNCS111525 

**Published:** 2025-03-14

**Authors:** Aman Pal, Emmanuel Aydin-Ghormoz, Swati Mehta, MJ Hajianpour, Emily Gaine, Muhammad Ali Zia, Elie Tannous, Andrea Lightle, Krishnakumar Hongalgi

**Affiliations:** 1 Department of Medicine,; 2 Department of Medicine, Division of Nephrology & Hypertension Care,; 3 Division of Medical Genetics and Genomics, Albany Med Health System, Albany Medical College, Albany, NY, USA,; 4 University Hospital Galway, Galway, Ireland, and; 5 Department of Pathology and Laboratory Medicine, Albany Medical Center, Albany, NY, USA

**Keywords:** TMA, complement-mediated TMA, aHUS, influenza virus, hemoptysis, plasmapheresis, eculizumab, genotype-phenotype correlation

## Abstract

Introduction: Thrombotic microangiopathy (TMA) is a pathological description which clinically presents with thrombocytopenia, microangiopathic hemolytic anemia (MAHA), and organ dysfunction. The etiology of TMA is broadly classified into four categories: primary hereditary, primary acquired, secondary, and infection associated. H1N1 influenza is a rare etiology of complement-mediated TMA (CM-TMA) with there being under 30 cases reported to date, and its odd presentation with hemoptysis making it a challenge to diagnose. Case presentation: We present a case of a Caucasian female in her 20s presenting to the hospital with a viral prodrome in setting of a new acute kidney injury (creatinine 8.2 mg/dL), thrombocytopenia (platelet count 14,000/mm^3^), and H1N1 influenza positive. She developed hemoptysis the next day, with no respiratory distress. Rheumatology work-up for antineutrophilic cytoplasmic antibodies (ANCA), anti-glomerular basement membrane (anti-GBM), and antiphospholipid syndrome (APS) antibodies was negative. CT chest was also negative for pulmonary hemorrhage. Plasma exchange was started empirically until ADAMTS13 activity returned normal (120%), and she was further commenced on eculizumab after an atypical hemolytic uremic syndrome (aHUS)/TMA/Complement 3 Glomerulopathy (C3G) gene panel was sent. Molecular studies revealed a splice site variant of MCP/CD46 gene, which was reiterated on a renal biopsy. The patient was counselled on the genetic results, including predisposition to future events and the importance of long-term eculizumab treatment. Discussion: CM-TMA is a consequence of alternative pathway dysregulation, commonly associated with genetic mutations which could phenotypically be unmasked by infections, such as influenza virus. Conclusion: Our case highlights the importance of keeping a broad differential beyond classic pulmonary-renal syndromes in patients presenting with hemoptysis and TMA, while understanding the pathophysiology of infections unmasking genetic mutations in CM-TMA.

## Introduction 

**Figure 1.**
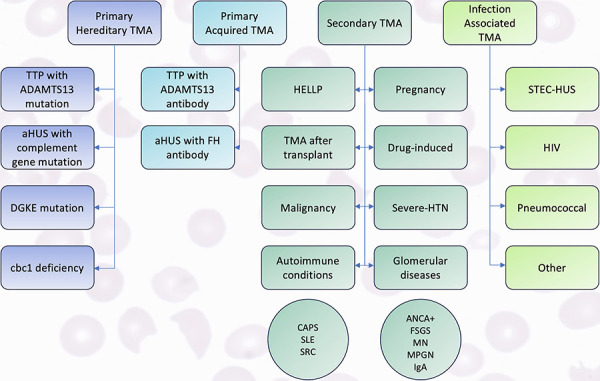
Extensive classification of thrombotic microangiopathy (TMA) broadly defined into four main categories: primary hereditary TMA, primary acquired TMA, secondary TMA, and infection-associated TMA. Data from Brocklebank et al. [1].

Thrombotic microangiopathy (TMA) is a pathological description which clinically presents with thrombocytopenia, microangiopathic hemolytic anemia (MAHA), and organ dysfunction. The classification of TMA has historically evolved from two broad categories, thrombotic thrombocytopenic purpura (TTP) and hemolytic uremic syndrome (HUS), to modern nomenclature using four categories of TMA: primary hereditary, primary acquired, secondary, and infection associated (Figure 1) [1]. Although there have been accelerating rates of discoveries in the pathogenesis, management, and outcomes of TMA, all etiologies have been unified by analogous clinical and pathological findings. 

## Case presentation 

A 20-year-old female presented as a transfer from an outside hospital with a 4-day history of hematuria, fatigue, intermittent fevers, and nausea/vomiting, on an unremarkable background history. 

A few days prior to presentation, the patient started experiencing a prodrome of fever, malaise, and myalgias, followed by development of a non-productive cough with mild dyspnea on exertion and nausea with limited oral intake. She tried to manage herself supportively with Tylenol and increased hydration during this time, however, endorsed multiple episodes of non-bilious, non-bloody emesis. Subsequently, her condition started to deteriorate when her symptoms progressed to right flank pain and gross hematuria. She initially attributed this to her menstrual period, which remained unlikely in the absence of blood on her pad. Additionally, the patient had an episode of hemoptysis but demonstrated no evidence of respiratory distress. Her past medical and surgical history were unremarkable, with no active medications or supplements. Her family history was non-contributory. She denied tobacco or illicit drug use, and occasionally drank alcohol. She lived with her significant other who had been ill with a “stomach bug” over the past week. Physical examination was remarkable for scleral icterus with conjunctival pallor and jaundice. 

She was referred to the emergency department by the outside hospital for further investigation of her thrombocytopenia and acute kidney injury (AKI). 

## Investigations 

On admission, the patient’s bedside urinalysis revealed 3+ protein, 1+ leukocyte esterase, positive nitrites, 3+ hemoglobin, marked bacteria with a spot urine protein-creatinine ratio of 29.2. Complete blood count (CBC) revealed a down trending hemoglobin to 9.6 g/dL with a platelet count of 14,000/mm^3^. Hemolysis work-up including lactate dehydrogenase (LDH) and haptoglobin were undetectable due to the specimen being hemolyzed, however, the peripheral blood smear showed schistocytes. Coagulation work-up was remarkable for fibrinogen 123 mg/dL and normal limits of prothrombin time (PT)/activated partial thromboplastin time (aPTT). Her complete metabolic panel (CMP) was significant for creatinine 8.85mg/dL and a bicarbonate of 19 mmol/L, with a high anion gap metabolic acidosis confirmed by a venous blood gas. Liver function tests revealed a bilirubin of 1.6 mg/dL, INR of 1, aspartate transaminase of 67 U/L, and alanine transaminase of 25 U/L. An extensive microbiological panel was obtained, with unremarkable results in the stool enteric pathogen panel and blood cultures, however, respiratory viral panel was positive for Influenza A (H1N1) pdm09 virus RNA. CT imaging of the chest revealed peribronchial nodularity and ground glass opacification in the left lung, indicative of airway inflammation and no evidence of pulmonary hemorrhage. A renal ultrasound, followed by a CT abdomen-pelvis were conducted and were negative for obstructing nephrolithiasis or discernible hydronephrosis. 

ADAMTS13 activity, inhibitor, and antibody levels were measured, and the aHUS/TMA/C3G genetics panel was sent. These tests were send-outs and results were not available during the first few days of admission. 

**Figure 2. Figure2:**
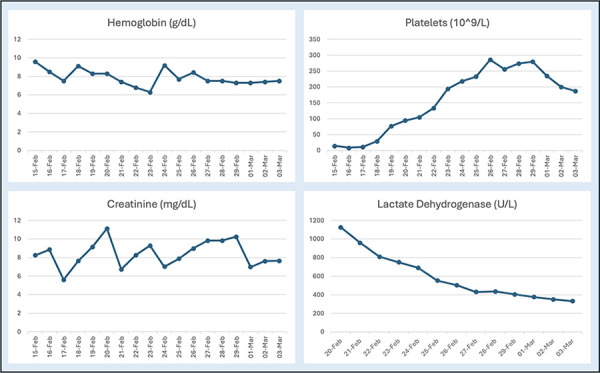
Trend of critical lab values during admission, plasma exchange (PLEX) and eculizumab treatments. Key dates included 15-Feb for admission, 16-Feb (day 1 PLEX), 17-Feb (day 2 PLEX), 18-Feb (day 1 eculizumab), 24-Feb (day 2 eculizumab), and 2-Mar (day 3 eculizumab).

In liaison with our rheumatology team, the patient underwent an extensive work-up for systemic autoimmune conditions consisting of antinuclear antibodies (ANA), anti-double-stranded deoxyribonucleic acid (anti-dsDNA) antibodies, anti-SSA (Ro) or anti-SSB (La) antibodies, ANCA, proteinase 3 antibodies (PR-3), myeloperoxidase (MPO) antibodies, serum protein electrophoresis (SPEP), anti-phosphatidylserine, and anti-GBM antibodies. APS antibodies including β-2-glycoprotein antibodies, anti-cardiolipin antibodies, lupus anticoagulant were also obtained. Serology for all autoimmune conditions were negative. C3 and C4 levels were 76.2 mg/dL and 20.7 mg/dL, respectively. [Fig Figure2]

## Differential diagnosis 

Given the subjective history of viral prodrome and hematuria, alongside objective findings of conjunctival pallor, jaundice, anemia, thrombocytopenia, and acute kidney injury, a TMA remained likely. The differentials for this phenomenon included TTP, Shiga toxin-producing *Escherichia coli* hemolytic uremic syndrome (STEC-HUS), complement-mediated TMA (CM-TMA), drug-induced TMA, metabolism-mediated TMA, or coagulation-medicated TMA. Systemic syndromes such as disseminated intravascular coagulation (DIC), infections, malignancy, preeclampsia, severe hypertension, and rheumatic diseases were also considered. 

## Treatment 

In the setting of her elevated PLASMIC score and significant thrombocytopenia, the decision was reached to start the patient on plasmapheresis until her ADAMTS13 results came back, with regular monitoring of CBC, CMP, LDH, haptoglobin, and fibrinogen. Given her rising creatinine, uremia, worsening acidosis, and poor urinary output, she underwent an emergent hemodialysis washout on the first day. She was also started on IV 1 g methylprednisolone for predicted course of 3 days while her autoimmune panel was pending. Additionally, she was commenced on broad-spectrum antibiotics given the developing pneumonia visualized on CT chest. 

After 2 days of treatment, the patient’s ADAMTS13 activity was found to be 120%, ruling out TTP and so the plasmapheresis was stopped. Her rheumatology work-up listed above was also negative, allowing the discontinuation of steroids. 

At this time, her presentation was concerning for HUS, and while we were awaiting the aHUS/TMA/C3G genetic panel, the patient was commenced on eculizumab 900 mg, once weekly × 4 doses, for presumable CM-TMA in the setting of a viral infection. She received her meningococcal vaccines prior to administration of this new regimen, and was placed on prophylactic ciprofloxacin. 

**Figure 3.**
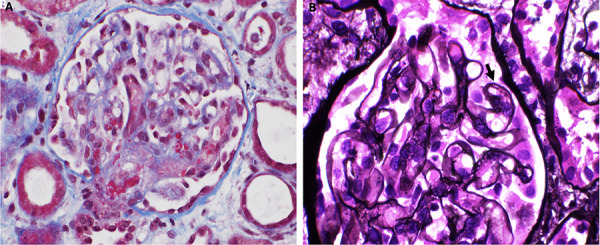
Histopathology from kidney biopsy: A: Arteriolar and mesangial fibrin with entrapped schistocytes (Masson’s trichrome stain, × 400). B: Segmental duplication of the basement membranes (arrow) (Jones methenamine silver stain, × 600).

The genetic panel noted the patient to have a heterozygous splice site variant (chr1:207757204, c.288+2T>G) 2 bp downstream from the end of exon 2 of MCP/CD46. Unfortunately, despite the extensive work-up and multiple interventions including intermittent dialysis, her renal function did not improve and she underwent a kidney biopsy after a couple days. This revealed focal acute on chronic TMA characterized by endothelial dwelling with entrapped schistocytes, hilar fibrin thrombi, arteriolar fibrinoid necrosis, and segmentally double-contoured glomerular capillary loops (Figure 3). 

## Outcome and follow-up 

Subjectively, our patient had marked improvement in her symptoms with complete resolution of her flu and hematuria within a few weeks of treatment. She continued to receive intermittent transfusions to maintain a hemoglobin goal greater than 7, but her platelets were able to recover to normal limits. She had been closely working with physical and occupational therapy, to regain her strength back to baseline with great recovery. Although her urine output significantly improved after commencing treatment, her creatinine remained elevated with evidence of acidosis. A plan was made to place a permanent dialysis catheter and arrange for an outpatient hemodialysis unit near her house. Before these arrangements could be finalized, the patient left against medical advice and from our records, she had only received 3 out of the 4 recommended doses of eculizumab. 

## Discussion 

Our patient who presented with MAHA, thrombocytopenia, and AKI was imperatively started on plasma exchange due to concerns for TTP, the commonest cause of TMA. Her viral prodrome, nausea and vomiting, and poor oral intake raised concerns for a pre-renal etiology of AKI, which remained unlikely after a lack of response to intravenous fluids. Another differential to consider was a urinary tract infection with the constitutional symptoms described above alongside positive urinalysis findings. However, the lack of significant colonization of bacteria in urine and blood cultures made this differential unlikely. Given the patient’s episode of hemoptysis, concerns for classic pulmonary-renal syndromes like Goodpasture’s disease and small vessel vasculitides were considered, but these were ruled out with negative serologies. While isolated cases of diffuse alveolar hemorrhage in TMA exist, this was not seen on imaging in the patient. Instead, the hemoptysis and gross hematuria were more likely due to severe thrombocytopenia secondary to TMA. The patient’s ADAMTS13 level was found to be 120%, and a negative stool enteric pathogen panel ruled out common causes of TMA, TTP, and STEC-HUS. As a result, the patient was started on eculizumab for aHUS while awaiting the results of her genetic screening. 

**Figure 4.**
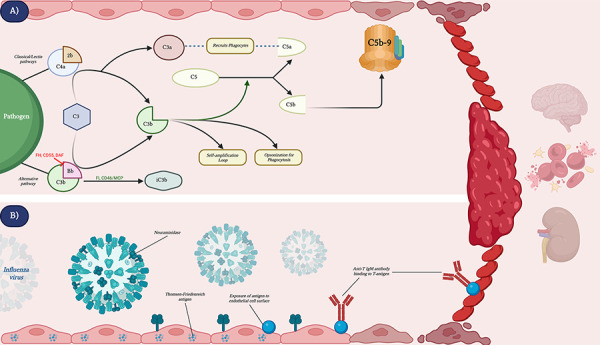
A: Pathophysiology of complement-mediated thrombotic microangiopathy from pathogen-initiation of the three complement pathways to activation of membrane attack complex, predisposing patients to clinical syndromes. B: Pathophysiology of proposed second trigger, influenza virus, further exacerbating endothelial damage and promoting thrombosis. Created in BioRender. Ali, O. (2025) https://BioRender.com/l22d977.

CM-TMA, also referred to as aHUS, is a consequence of alternative pathway dysregulation. The complement system is part of the innate immune system involved in killing pathogens and is composed of three main pathways: classical, lectin, and alternative. All pathways share the common steps of generating C3 convertase, an enzyme which is responsible for the cleavage of C3 into C3a and C3b. C3a is released into the circulation where it recruits neutrophils and monocytes to the site of infection. On the other hand, C3b is involved in opsonization for phagocytosis, self-amplification, and binding to the pre-existing C3 convertase to form C5 convertase. The C5 convertase cleaves C5 into C5a, a complement which recruits phagocytes, and C5b, a complement responsible for the production of the membrane attack complex (MAC). This complex further goes on to form pores in pathogen cell membranes, causing the cell to lyse. When this complement activation affects endothelial cells, it can result in CM-TMA. However, in order to prevent host cell destruction, there are a number of downregulatory complement proteins that prevent overstimulation of the pathway. For starters, decay-accelerating factor (DAF) and CD55 are the first downregulatory proteins which compete with factor B binding to C3b, reducing the formation of C3 convertase in the alternative pathway. Factor H (FH) is another plasma protein that outcompetes factor B by binding to C3b on host cells, preventing activation on them. Another mechanism through which host cells are protected is through the conversion of C3b into its inactive form. This is achieved with plasma protease, factor I (FI), and membrane cofactor proteolysis (MCP), also known as CD46. Genetic mutations or acquired antibodies against any of these factors can result in disruption of the physiological defense mechanisms, and further promote cell damage [2] (Figure 4A). 

Up to 60% of all aHUS cases have demonstrated the role of genetic mutations and acquired antibodies [3]. In a study conducted by Fremeux-Bacchi et al. [4], mutation frequency was assessed in 214 cases of aHUS, with 27.5% of patients having complement factor H mutations, 9.3% positive for MCP mutations, and 8.4% positive for complement factor I mutations. Another study by Marina et al. [5] looked at the genetic screening in 273 patients with aHUS and reported mutation frequency rates of 24%, 7%, and 4% for complement factor H, MCP, and complement factor I, respectively. Although these mutations are fairly prevalent, not all individuals present with disease as these mutations are thought to have incomplete penetrance. The disease penetrance is age-related, with up to 64% of patients having an acute episode by the age of 70 [6]. Additionally, these mutations are considered to be predisposing factors to disease, with there being secondary triggers such as pregnancy or infection which would unmask the latent complement defect [1]. Unlike the genetic mutations, acquired autoantibodies is a fairly newer studied entity with the first case of antibodies against factor H being reported in 2005 and further studies demonstrating the prevalence of anti-FH antibodies in up to 20% of aHUS cases [3]. 

One of the proposed mechanisms that can unmask these latent complement deficits include infections, such as influenza virus. In a literature review conducted by Bitzan and Zieg [7], 25 cases of influenza-associated TMA were identified and among this cohort, 83% were found to be influenza A-positive. Its pathophysiology is thought to be related to the production of neuraminidase (NA), which is also a proposed mechanism of pneumococcal HUS [1, 7]. This enzyme is known to cleave glycoproteins, exposing the hidden Thomsen-Friedenreich antigen to endothelial cell and platelet surfaces. Subsequent binding to anti-T IgM antibodies results in platelet aggregation and endothelial injury [8, 9] (Figure 4B). Other proposed mechanisms include direct damage to endothelial cells by the virus, increased formation of platelet-monocyte aggregates and activation of integrin on platelets [7, 10]. Finally, since complement proteins are an integral part of the innate immune system, their activation during inflammatory and infective processes results in increased formation of MAC as well [7]. Our patient was found to be H1N1 positive. In addition to her pre-existing CD46 splice site mutation, this recent ailment most likely acted as a trigger to induce CM-TMA through the mechanisms described above. 

In the pre-eculizumab era, the clinical outcomes of CM-TMA were poor with roaring recurrence rates of 68% and 5-year post-transplantation death-censored graft survival rates of 51% [11]. In 2011, the United States Food and Drug Administration (FDA) approved a humanized monoclonal antibody, eculizumab, for the treatment of aHUS in pediatric and adult cohorts. This novel therapeutic agent works by inhibiting the cleavage of C5 into C5a and C5b, preventing the formation of MAC as illustrated in Figure 4A. Its efficacy was proven in its early stages as in a prospective study by Legenedre et al. [12]; two trials were conducted in patients who were 12 years of age and older with aHUS over a course of 62 and 64 weeks, respectively. These results demonstrated a mean increase in platelet count of 73 ×10^9^/L (p < 0.001) from baseline to week 26 in trial 1, and an 80% rate of TMA event-free status demonstrated in trial 2 [12]. Another single-arm phase 2 trial conducted by Fakhouri et al. [13], analyzed complete TMA response after 26 weeks of treatment and highlighted the improvements in renal, hematological, and quality of life manifestations. Whether it is an acutely progressive TMA or a longer duration associated with chronic kidney disease, a year 2-year analysis conducted by Licht et al. [14], demonstrated therapeutic benefits, with hematologic normalization seen in the former set of cases and a TMA event-free status seen in majority of the latter cases. Despite the clinical evidence demonstrated in each of the studies, the duration of treatment remains uncertain due to the high rates of relapse after withdrawal of treatment. In a study of 38 patients with aHUS who stopped eculizumab after a median treatment of 17.5 months, 31% (12 patients) relapsed after a median follow-up of 22 months. Relapse rates varied by genetic variant: 72% of patients with complement factor H variants (8/11), 50% with membrane cofactor protein variants (4/8), and 0% of patients with no rare variant (0/16) relapsed [15]. The prognosis of CM-TMA is significantly influenced by genetic mutations, particularly in complement genes such as CFH, MCP/CD46, and C3. Another systematic review conducted by Acosta-Medina et al. [16] found an overall relapse rate of 29.6% in patients who discontinued eculizumab after a median follow-up of 23 months, with the highest relapse rates observed in those with CFH and MCP/CD46 mutations, especially in the canonical splice regions. Additionally, younger age and a history of renal transplantation were independent risk factors for relapse. While eculizumab discontinuation can be appropriate for some patients, caution is warranted in those with high-risk genetic variants, as they are more likely to experience relapse. Genetic testing plays a crucial role in identifying these high-risk patients and informing treatment decisions, with lifelong therapy recommended for those at higher risk of recurrence [16]. 

Although the current license is for lifelong treatment, there has been evidence of rapid reintroduction (< 48 hours) of eculizumab leading to effective hematologic remission [13]. It is generally well tolerated with minimal risks, but due to its MAC-inhibiting properties, vaccination against encapsulated microbes (including *Neisseria meningitidis*, serogroups ACWY and B) should occur at least 2 weeks before starting treatment; if eculizumab is given prior to vaccination, the FDA recommends adding antimicrobial prophylaxis for at least 2 weeks after vaccination [17]. 

## Conclusion 

Our case emphasizes the importance of maintaining a broad differential diagnosis in patients with hemoptysis and TMA, while also highlighting the critical role of understanding the pathophysiology of CM-TMA. Infections, such as H1N1 influenza, can unmask underlying genetic mutations that lead to dysregulation of the alternative complement pathway. This case underscores the value of genetic testing, the need for targeted treatments like eculizumab, and the significance of understanding how infections can trigger or exacerbate genetic predispositions to TMA. 

## Data availability 

Access to data is permitted with the authors’ permission. 

## Consent 

Written consent was not obtained from the patient. 

## Authors’ contributions 

AP, EAG, SM, and KH participated in data collection. AP and EG wrote the manuscript and made the figures. EAG, SM, KH, and MH corrected the manuscript. ET and AL provided biopsy results. SM and KH supervised the project. All authors take responsibility that this study has been reported honestly, accurately, and transparently, and accept accountability for the overall work by ensuring that questions pertaining to the accuracy or integrity of any portion of the work are appropriately investigated and resolved. 

## Funding 

None. 

## Conflict of interest 

The authors declare that they have no conflict of interest.
